# The YBX3 RNA-binding protein posttranscriptionally controls *SLC1A5* mRNA in proliferating and differentiating skeletal muscle cells

**DOI:** 10.1016/j.jbc.2023.105602

**Published:** 2023-12-29

**Authors:** Silina Awad, William Skipper, William Vostrejs, Kendall Ozorowski, Kristen Min, Liva Pfuhler, Darshan Mehta, Amy Cooke

**Affiliations:** Biology Department, Haverford College, Haverford, Pennsylvania, USA

**Keywords:** YBX3, SLC1A5, SLC7A5, SLC3A2, amino acid transport, RNA-binding protein, C2C12 myoblasts, 3′ untranslated region (UTR)

## Abstract

In humans, skeletal muscles comprise nearly 40% of total body mass, which is maintained throughout adulthood by a balance of muscle protein synthesis and breakdown. Cellular amino acid (AA) levels are critical for these processes, and mammalian cells contain transporter proteins that import AAs to maintain homeostasis. Until recently, the control of transporter regulation has largely been studied at the transcriptional and posttranslational levels. However, here, we report that the RNA-binding protein YBX3 is critical to sustain intracellular AAs in mouse skeletal muscle cells, which aligns with our recent findings in human cells. We find that YBX3 directly binds the solute carrier (*SLC*)*1A5* AA transporter messenger (m)RNA to posttranscriptionally control SLC1A5 expression during skeletal muscle cell differentiation. YBX3 regulation of *SLC1A5* requires the 3′ UTR. Additionally, intracellular AAs transported by SLC1A5, either directly or indirectly through coupling to other transporters, are specifically reduced when YBX3 is depleted. Further, we find that reduction of the YBX3 protein reduces proliferation and impairs differentiation in skeletal muscle cells, and that YBX3 and SLC1A5 protein expression increase substantially during skeletal muscle differentiation, independently of their respective mRNA levels. Taken together, our findings suggest that YBX3 regulates AA transport in skeletal muscle cells, and that its expression is critical to maintain skeletal muscle cell proliferation and differentiation.

Skeletal muscle constitutes nearly 40% of the healthy adult body mass, and contributes significantly to multiple functions such as movement, metabolism, and a critical reservoir of amino acids and sugars like glucose (1, 2). It has become increasingly clear that posttranscriptional control is critical for development, growth, and repair in the skeletal muscle system, which demonstrates the need to understand posttranscriptional regulation in skeletal muscles (2). These emerging findings have led to studies aimed at developing RNA-based therapies for multiple debilitative muscular disorders including myotonic dystrophy (3), oculopharyngeal muscular dystrophy (4), and spinal muscular atrophy (5).

The Y-box proteins, YBX1 and YBX3, are nucleic acid binding proteins that have overlapping mRNA targets and regulatory functions (6, 7, 8). These proteins are broadly expressed during embryonic development, but upon birth, YBX3 is expressed predominantly in the heart, skeletal muscle, blood vessels, and testis (9, 10, 11). Interestingly, while both YBX1 and YBX3 mRNAs are highly expressed in the human skeletal muscle, only YBX3 protein is present offering a system to study YBX3 regulation independent of YBX1 (12). In C2C12 mouse myoblast cells, altered YBX3 expression resulted in myoblast differentiation defects through transcriptional regulation of myogenin; however, posttranscriptional control mediated by YBX3 has yet to be investigated (9).

We recently showed that in human cervical cancer cells, YBX3 stabilizes the mRNAs of the L1 amino acid transporter system comprised of two solute carrier (SLC) proteins, SLC7A5 and SLC3A2 (13, 14). Upon YBX3 knockdown (KD), the mRNA levels of the *SLC7A5* and *SLC3A2* transporter mRNAs significantly decreased, which reduced their protein levels and the influx of the amino acids imported by the complex (14). Importantly, skeletal muscle cells require the import of amino acids to maintain as a reservoir of amino acids in muscle protein and for muscle mass (1), yet posttranscriptional regulation of SLC transporters has not been explored in this system. SLC amino acid transporter expression is known to be highly coordinated and respond to extracellular stimuli in skeletal muscles (15, 16, 17, 18). For example, after resistance exercise and/or amino acid ingestion there is a marked increase in the mRNA and protein levels of SLC7A5, SLC3A2, SLC38A2, SLC36A1, and SLC7A1 in skeletal muscles (19, 20). This response has been implicated in amino acid exchange needed for protein synthesis as muscles remodel (19). Other amino acid transporters critical in muscle cell includes the glutamine transporter, SLC1A5 (21). Glutamine, a critical amino acid for skeletal muscle cell proliferation and differentiation, makes up approximately 50 to 60% of the total amino acid pool in skeletal muscle tissue (21, 22, 23).

We set out to identify genes that are posttranscriptionally controlled by YBX3 in skeletal muscle cells, in particular to determine if YBX3 directly binds and regulates SLC amino acid transporter mRNAs. We first depleted YBX3 in C2C12 mouse skeletal muscle cells during differentiation, and then performed transcriptomic analysis to identify candidates. As our previous results demonstrate YBX3 directly binds and regulates transporter mRNAs in human cells, we focused our analysis on amino acid transporters (14). Our RNAseq data revealed reduced *SLC1A5* mRNA in cells depleted of YBX3; however, *SLC7A5* and *SLC3A2* were not reduced as previously observed in HeLa cells (14). We found that YBX3 directly binds *SLC1A5* mRNA, and that YBX3 depletion specifically reduces SLC1A5 mRNA and protein expression during muscle cell differentiation. Importantly, upon YBX3 depletion, we find that intracellular amino acids directly (glutamine) and indirectly (branched chain amino acids) transported by SLC1A5 are reduced, and that YBX3 depletion impacts skeletal muscle proliferation and differentiation. Furthermore, we find a marked increase in YBX3 and SLC1A5 protein expression independent of changes in their mRNA levels. Taken together, our findings suggest that YBX3 posttranscriptional control is critical to maintain amino acid homeostasis, and that YBX3 expression is needed for skeletal muscle proliferation and differentiation.

## Results

### Identification of genes affected by YBX3 depletion in skeletal muscle cells

**Figure 1:**
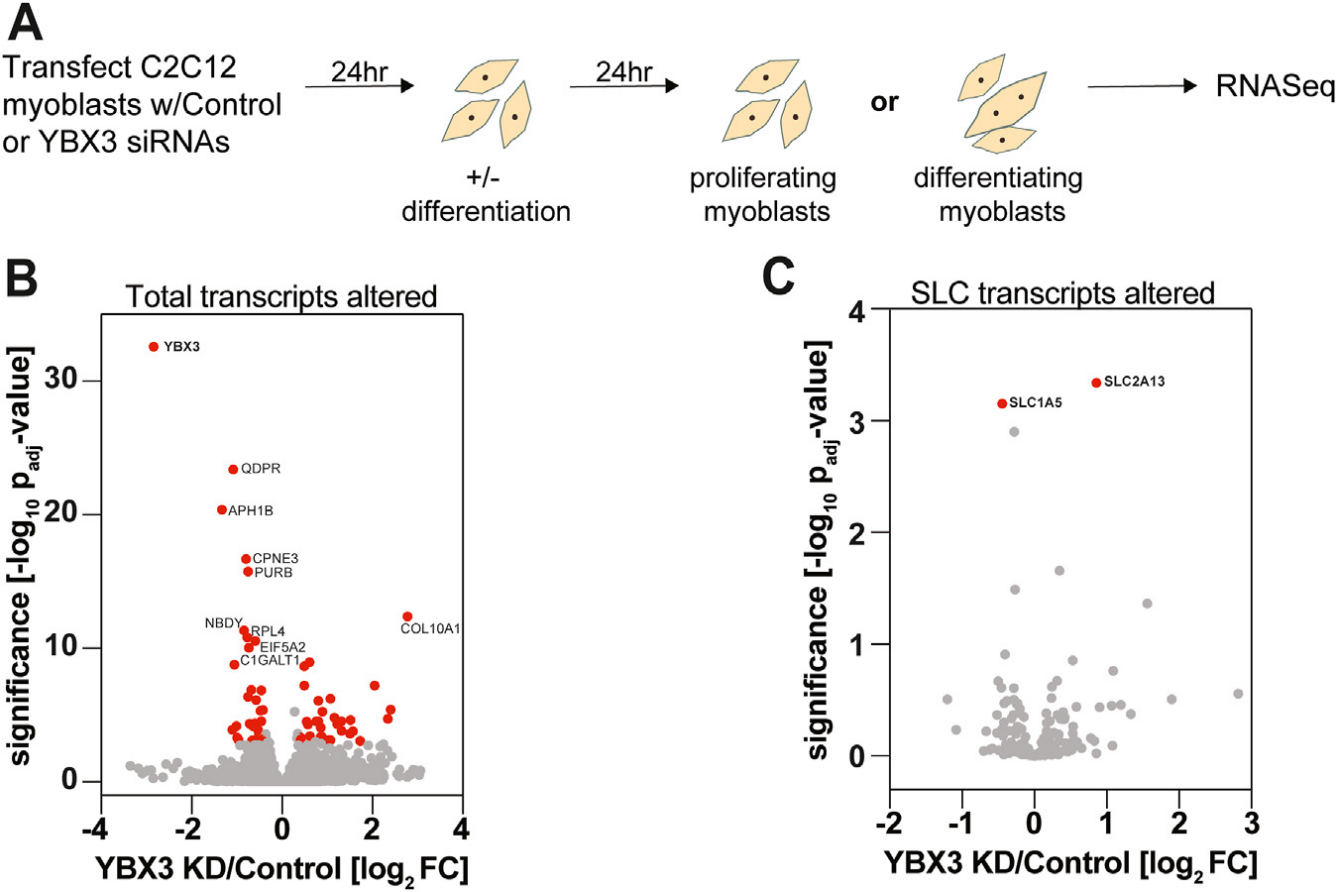
**Transcriptomic changes elicited by YBX3 knockdown in skeletal muscle cells.***A*, a schematic of an approach to identify genes affected by YBX3 knockdown (KD) in C2C12 skeletal muscle cells. Cells transfected with either nontargeting or YBX3 targeting small interfering RNAs (siRNAs) were differentiated or not 24 h (hr) posttransfection, followed by an additional 24 h incubation before samples were collected to assess changes in transcript levels *via* high-throughput RNA sequencing. *B*, volcano plot of altered transfect from cells in (*A*) that combines results from proliferating and differentiating myoblast results. RNAs were classified as significant (FC > ±1.3 and p-adjusted 0.001; *red dots*) or nonsignificant (*gray dots*). Labels of significantly altered transcripts with a p-adj of 0.0001 indicated. In total, 55,642 RNAs identified with 34 reduced and 34 increased upon YBX3 KD *versus* control KD. *C*, volcano plot of all *SLC* transcripts detected in RNA sequencing from (*B*). FC, fold change; SLC, solute carrier.

To identify genes whose expression is controlled by YBX3, we used high-throughput RNAseq in C2C12 mouse skeletal muscle cells depleted of YBX3 *via* siRNA. As YBX3 has known roles in differentiation (10, 24), we measured changes in transcript levels in myoblasts before (proliferating) and one day after differentiation initiation (differentiating) (Fig. 1*A*), which allowed for cells to be harvested concurrently at 48 h post siRNA transfection. As expected, RNAseq data revealed that YBX3 mRNA was efficiently depleted (Figs. 1*B* and S1, *A* and *B*). In total, 55,642 transcripts were identified between the two time points with 68 transcripts changed in expression upon YBX3 depletion (absolute fold change > 1.3 with adjusted *p*-value <0.001, Fig. 1*B*). Of the identified transcripts, 34 had increased expression and 34 were reduced in their expression (Fig. 1*B*). The changes in expression upon YBX3 KD did not differ between proliferating and differentiating myoblasts (Fig. S1, *A*–*C*); therefore, we combined the two time points (Fig. 1*B*). The majority of changes were among low abundant transcripts with only a few exceptions, *e.g.*, *RPL10* (Fig. S1*D*). These results suggest that YBX3 influences the expression of a select number of RNAs in mouse skeletal muscle cells.

### YBX3 reduces SLC1A5 expression in mouse skeletal muscle cells

**Figure 2:**
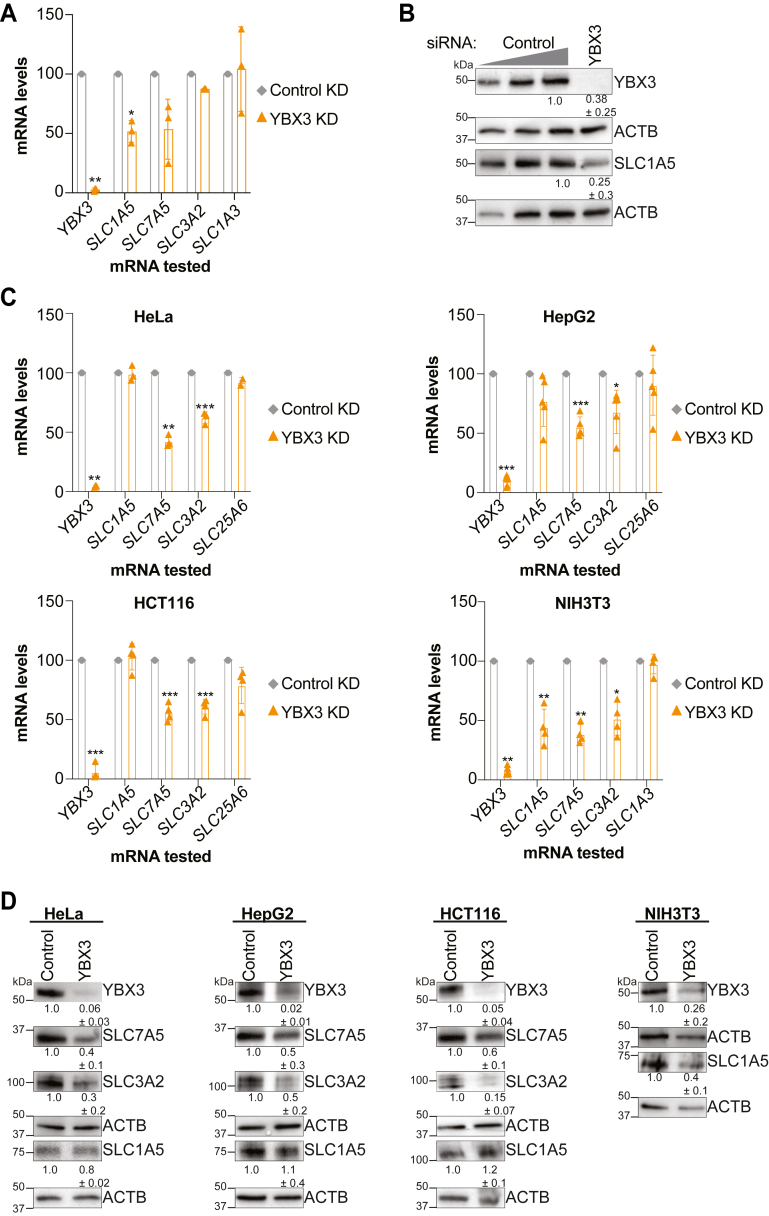
**YBX3 depletion reduces SLC1A5 expression in C2C12 and NIH3T3 mouse cells but not in HeLa, HepG2, and HCT116 human cell lines.***A*, reverse transcription quantitative polymerase chain reaction (RT-qPCR) of relative mRNA levels after YBX3 KD *versus* control KD in C2C12 cells 1-day post differentiation initiation. *B*, immunoblot analysis of protein levels for YBX3 and SLC1A5 expression along with actin loading control (ACTB) in differentiating myoblasts in C2C12 cells transfected with nontargeting or YBX3 targeting siRNAs. Lysates from cells treated with nontargeting siRNAs are loaded at 25%, 50%, and 100%. Protein quantifications (n = ≥3) and molecular mass markers are indicated. *C* and *D*, RT-qPCR analysis of relative mRNA levels (*C*) or immunoblot analysis of protein levels (*D*) after YBX3 depletion from indicated cell lines. Protein quantifications (n = ≥2) and molecular mass markers are indicated. All cells collected 48 h after siRNA transfection. ∗∗∗*p* < 0.001, ∗∗*p* < 0.01 and *p* < 0.05 with paired Student’s *t* test, n = ≥3. All RT-qPCR values normalized to *UBN1* (mouse cells) or *GUSB* (human cells) mRNA levels and protein levels to loading control ACTB. SLC, solute carrier.

As skeletal muscle cells are a critical reservoir of amino acids in proteins (1, 2) and YBX3 posttranscriptionally regulates the L1 amino acid transporters mRNAs *SLC7A5* and *SLC3A2* in human cells (14), we focused on changes in SLC mRNAs (Fig. 1*C*). Surprisingly, we found that YBX3 depletion in C2C12 does not alter *SLC7A5* and *SLC3A2* mRNA levels, but it does significantly reduce a neutral amino acid transporter (*SLC1A5* AKA *ASCT2*) and increase a novel gamma-secretase associated protein (*SLC2A13*) (Fig. 1*C*). Therefore, we focused on SLC1A5 expression as this protein imports neutral amino acids, in particular glutamine, which is critical during bone development in mice and skeletal muscle cell proliferation and differentiation (22, 23, 25). We first assessed the steady-state mRNA of *SLC1A5* in proliferating (Fig. S2) and differentiating myoblasts (Fig. 2*A*). In agreement with the RNAseq data, we found that *SLC1A5* mRNA is reduced using reverse transcription quantitative polymerase chain reaction (RT-qPCR), whereas the levels of *SLC7A5, SLC3A2*, and *SLC1A3* were not significantly altered in YBX3 KD C2C12 cells (Figs. 2*A* and S2*A*). Next, we tested if the changes in mRNA result in altered SLC1A5 protein expression in differentiating myoblasts. Immunoblot analysis revealed that SLC1A5 levels also decrease upon YBX3 KD, while actin (ACTB) was unaffected by YBX3 depletion (Fig. 2*B*). As expected, YBX3 was efficiently KD at both the mRNA (Figs. 2*A* and S2*A*) and protein levels (Fig. 2*B*). Additionally, the individual siRNAs used to deplete YBX3 resulted in similar reduction of YBX3 and SLC1A5 protein expression in differentiating myoblasts (Fig. S2*B*). Together, these data suggest that YBX3 controls the expression of SLC1A5 in mouse skeletal cells through the regulation of *SLC1A5* mRNA levels, which could be due to changes in transcription or mRNA stability or a combination of these processes.

### YBX3 regulates SLC1A5 in mouse cells but not in human cell lines

Next, we wanted to assess if YBX3 regulates SLC1A5 expression in human cells as our enhanced cross-linking, and immunoprecipitation (IP) data in HeLa show that YBX3 directly crosslinks to *SLC1A5* (14). SLC1A5 mRNA and protein levels are unaffected by YBX3 depletion in HeLa cells, while SLC7A5 and SLC3A2 mRNA and protein are reduced as expected (14) (Fig. 2, *C* and *D*). To assess if this is conserved between mouse and human lines, we tested the effects of YBX3 KD on these SLCs in other human (HCT116 and HepG2) and mouse cells (NIH3T3). We found that YBX3 KD reduces SLC1A5 mRNA and protein expression in NIH3T3 cells but not in any of the human cell lines, while SLC3A2 and SLC7A5 expression is affected in all human cell lines and NIH3T3 (Fig. 2, *C* and *D*). Taken together, these data suggest that YBX3 controls SLC1A5 expression *via* its mRNA in mouse cells but not in human cell lines.

### YBX3 and SLC1A5 protein expression increase during skeletal muscle differentiation

**Figure 3:**
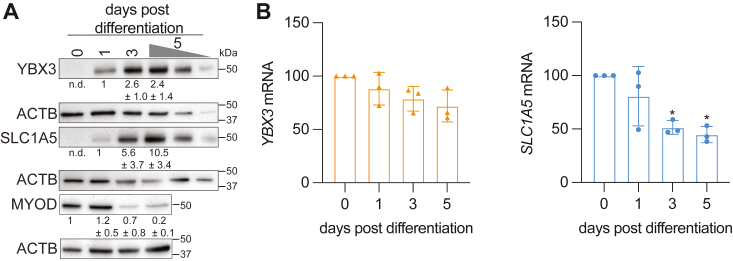
**YBX3 and SLC1A5 protein expression increase throughout differentiation independent of changes in mRNA.***A*, immunoblot analysis of protein levels for YBX3 and SLC1A5 expression along with the loading control, ACTB, and differentiation marker, MYOD. Lysates from cells that differentiated for 5 days are loaded at 100%, 50%, and 25% except for MYOD and its respective ACTB loading control. Protein quantifications (n = ≥3) and molecular mass markers are indicated, n.d. = not detected. *B*, RT-qPCR of *YBX3* and *SLC1A5* relative mRNA levels across 5 days of differentiation. ∗ = *p* < 0.05 with paired Student’s *t* test, n = ≥3. RT-qPCR values normalized to *UBN1* mRNA levels. RT-qPCR, reverse transcription quantitative polymerase chain reaction; SLC, solute carrier.

YBX3 and SLC1A5 protein expression was difficult to assess in myoblasts before differentiation initiation, that is, in proliferating myoblasts, yet both mRNAs were present (Figs. S1 and S2). Therefore, we next wanted to assess how these proteins were expressed throughout skeletal muscle cell differentiation. Surprisingly, there was a marked increase in YBX3 and SLC1A5 protein expression 1-day post initiation of differentiation, and a ∼2.5-fold and 5-fold increase of YBX3 and SLC1A5, respectively, by three days post initiation of differentiation (Fig. 3*A*). While YBX3 protein levels remained relatively constant between three and five days post initiation of differentiation, SLC1A5 expression increased by nearly 2-fold from three to five days post differentiation (Fig. 3*A*). Importantly, a well-established skeletal muscle differentiation marker (26), MYOD, decreases upon differentiation, as expected (Fig. 3*A*). We next assessed whether the increase in YBX3 and SLC1A5 protein expression is a result of higher levels of their respective mRNAs. The levels of *YBX3* mRNA did not significantly increase throughout skeletal muscle differentiation (Fig. 3*B*), while the levels of *SLC1A5* stayed relatively the same 1-day postdifferentiation initiation and then significantly decreased (Fig. 3*B*). The abundance of *SLC7A5* and *SLC3A2* mRNAs did not significantly change across differentiation, while the *SLC1A3* mRNA significantly decreased throughout differentiation (Fig. S3). These data suggest that the increase in YBX3 and SLC1A5 protein levels are not due to increases in the transcription rate or stability of their mRNAs.

### YBX3 interacts with SLC1A5 mRNA in skeletal muscle

**Figure 4:**
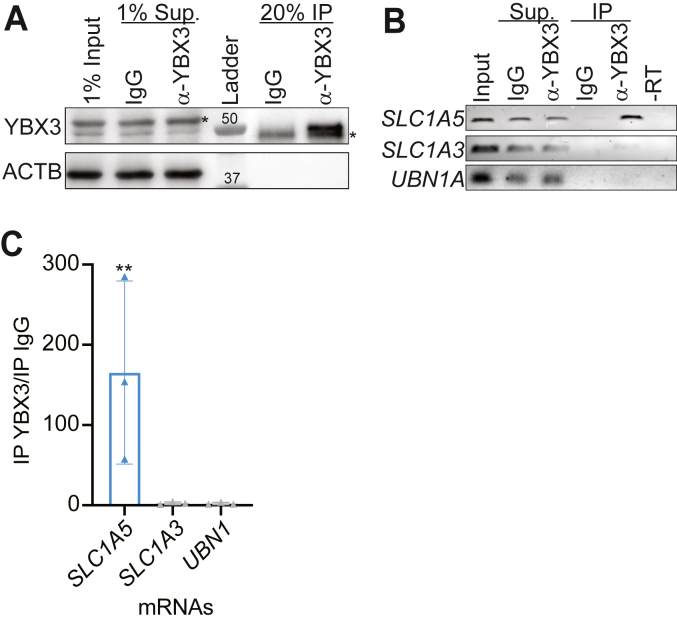
**YBX3 interacts with *SLC1A5* mRNA in skeletal muscle cells.***A*, immunoblot analysis of YBX3 in immunoprecipitation (IP) assay. YBX3 IP was performed alongside total rabbit IgG control IP with the same cell lysate from C2C12 1-day post differentiation initiation. Percent of the of the total lysate (input), unbound supernatant (sup) or IP samples from the YBX3 or rabbit IgG control IPs were assessed. Nonspecific bands indicated (∗) and actin (ActB) serves as a loading control. Protein quantification and molecular mass marker indicated. *B*, end point PCR of indicated mRNAs from each sample in (*A*), n = >3. *C*, RT-qPCR of indicated mRNA from IP samples plotted as a ratio of YBX3 IP/Control IP. *SLC1A3* and *UBN1A* are negative controls. RT-qPCR values normalized to input mRNA levels. ∗∗ = *p* < 0.01 with paired Student’s *t* test, n = 3. IgG, immunoglobulin G; RT-qPCR, reverse transcription quantitative polymerase chain reaction; SLC, solute carrier.

In human cells, YBX3 directly binds SLC mRNAs to control their levels (14). To determine if YBX3 interacts with *SLC1A5* mRNA in skeletal muscle, we performed a coimmunoprecipitation (co-IP) assay in C2C12 1 day after differentiation initiation. YBX3 was effectively pulled-down in the co-IP compared to controls (rabbit immunoglobulin G (IgG) and β-actin) (Fig. 4*A*). We analyzed the RNAs that were pulled-down with YBX3 using end point PCR and RT-qPCR (Fig. 4, *B* and *C*, respectively). As previously seen in human cells (14), we found that YBX3 interacts with the *SLC1A5* transcript; however, the control transcripts (*SLC1A3* and *UBN1A*) are not detected (Fig. 4*B*). We quantified the enrichment of *SLC1A5* mRNA bound by YBX3 using RT-qPCR, which revealed a significant enrichment in the YBX3 co-IP samples relative to the control IgG co-IP (Fig. 4*C*), while there was no enrichment of the *SLC1A3* or *UBN1* (Fig. 4*C*). Surprisingly, YBX3 binds both *SLC7A5* and *SLC3A2* mRNAs in mouse skeletal muscle cells (Fig. S4). These results demonstrate that YBX3 interacts with specific *SLCs* in mouse skeletal muscle cells. Combined with the YBX3 KD data, these results suggest that YBX3 binds *SLC1A5* to posttranscriptionally control SLC1A5 mRNA and protein levels.

### YBX3 regulation of SLC1A5 mRNA is mediated *via* its 3′ UTR

**Figure 5:**
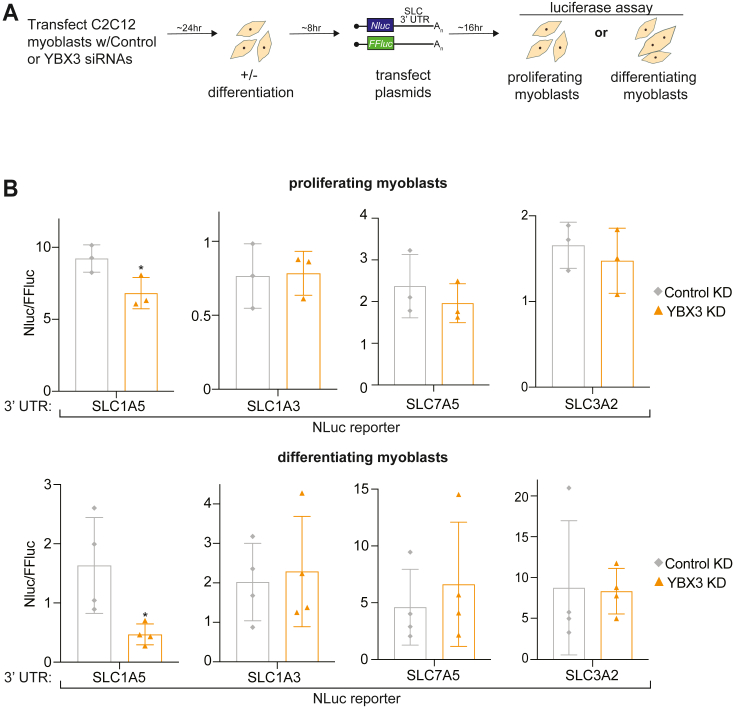
***SLC1A5* 3′ UTR is sufficient for YBX3 regulation.***A*, a schematic of an approach to determine if the *SLC1A5* 3′ untranslated region (UTR) is sufficient sufficiency for YBX3-mediated regulation in C2C12 cells. Cells transfected with either nontargeting or YBX3 siRNAs were either induced or not induced to differentiate 24 h after transfection followed by luciferase reporter plasmid transfection 8 h after cells were either differentiated or not and luciferase levels were assayed ∼16 h later. NanoLuc (NLuc) plasmids contained indicated *SLC* 3′ UTR; FFLuc plasmid with a B-globin 3′ UTR was used as a transfection and normalization control. *B*, dual luciferase assay of normalized NLuc reporter expression (NLuc/FFLuc) with the indicated *SLC* 3′ UTRs (*SLC1A5*, *SLC1A3*, *SLC7A5*, and *SLC3A2*) in proliferating (*top row*) or differentiating (*bottom row*) myoblasts. All dual luciferase assay data are displayed as single points, mean ±SD. ∗*p* < 0.05 with paired Student’s *t* test, n = 3 (proliferating myoblasts) or n = 4 (differentiating myoblasts) FFLuc, firefly luciferase; SLC, solute carrier.

In HeLa cells, the 3′ UTR of *SLC7A5* is necessary and sufficient for YBX3 to modulate SLC7A5 expression (14). Therefore, we sought to determine if the 3′ UTR of *SLC1A5* is required for YBX3 to posttranscriptionally control SLC1A5 expression in C2C12. We cloned the 3′ UTR of *SLC* transcripts (*SLC1A5*, *SLC7A5*, *SLC3A2*, and *SLC1A3*) downstream of the *NanoLuc* (*nluc*) reporter RNA. Each NLuc-SLC 3′ UTR was cotransfected with a *firefly luciferase* (*ffluc*) plasmid into YBX3 depleted C2C12 cells followed by luciferase analysis as a proxy for changes in expression (Fig. 5*A*). We found that the presence of the *SLC1A5* 3′ UTR is sufficient to significantly reduce NLuc activity in both proliferating and differentiating myoblasts, whereas the 3′ UTRs of the other *SLC* transcripts did not affect NLuc activity (Fig. 5*B*). Importantly, ffluc activity was unaffected by YBX3 KD when cotransfected with any of the NLuc-SLC 3′ UTR reporter plasmids in either proliferating or differentiating myoblasts (Fig. S5). Together, these data reveal that the *SLC1A5* 3′ UTR is sufficient for YBX3 to posttranscriptionally regulate SLC1A5 expression before and during differentiation.

### YBX3 regulates the intracellular levels of specific amino acids

**Figure 6:**
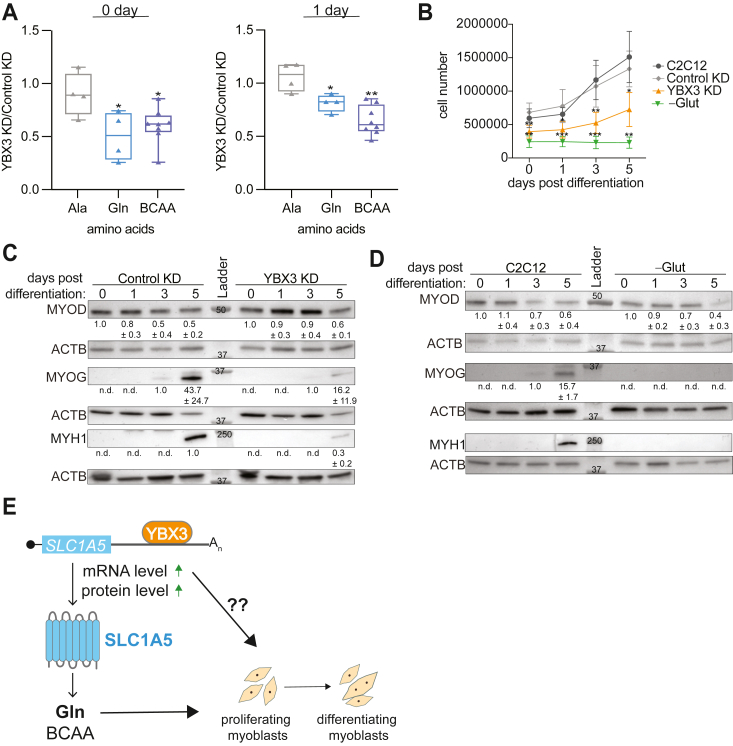
**YBX3 depletion specifically reduces intracellular glutamine and inhibits cell proliferation and differentiation.***A*, relative concentration of alanine (Ala), glutamine (Gln), or branched chain amino acids (BCAAs) in C2C12 cells transfected with nontargeting (control KD) or YBX3 siRNAs (YBX3 KD) before (0-days) or 1-day post differentiation (1-day). *Box plot* displays standard deviation and the *line* marks the median values for the replicates. Each point indicates a biological replicate, n = ≥3. ∗∗ = *p*-value <0.01 and ∗ = *p*-value <0.05 with paired Student’s *t* test. *B*, cell count of C2C12 cells transfected with nontargeting (control KD) or YBX3 (YBX3 KD) siRNAs and nontransfected cells with (-Glut) and without (C2C12) glutamine starvation using BioRad TC20 at 0, 1, 3, and 5-days post differentiation. ∗ = *p* < 0.05, ∗∗ = *p* < 0.01, ∗∗∗ = *p* < 0.001, n = ≥4. *C* and *D*, immunoblot analysis of protein levels for MYOD, MYOG, and MYH1 expression along with actin loading control (ACTB) at 0, 1, 3, and 5-days post differentiation in C2C12 cells (*C*) transfected with nontargeting or YBX3 targeting siRNAs or (*D*) nontransfected cells with and without glutamine starvation. Protein quantifications (n = ≥3) and molecular mass markers are indicated. *E*, schematic of model for YBX3 regulation of intracellular amino acid levels during differentiation through interaction and regulation of *SLC1A5* transcript. SLC, solute carrier.

SLC1A5 is a sodium dependent amino acid transporter that transports neutral amino acids, notably glutamine, across the plasma membrane (21). To determine if YBX3-mediated regulation of *SLC1A5* mRNA affects intracellular amino acid levels in C2C12 cells, we used a targeted approach of assessing intracellular glutamine across the initial days of C2C12 differentiation. In cells depleted of YBX3 by either the pool or a single YBX3 siRNA, glutamine levels were significantly reduced in proliferating (0-days) and differentiating (1-day) myoblasts, in comparison with control depleted cells (Figs. 6*A* and S6*A*). To determine if this reduction is specific to amino acids transported by the SLC1A5 we next assessed if YBX3 KD affects an amino acid not imported by SLC1A5. For this reason, we selected L-alanine, which is transported by the neutral amino acid transporter SLC1A4/ASCT-1 (27). In YBX3 depleted cells, there was no significant difference in the relative alanine levels compared to control depleted cells at any time point tested (Fig. 6*A*).

It has been observed that SLC1A5 import of glutamine can be coupled with the SLC7A5/SLC3A2 bidirectional transporter system to export glutamine and import branch chained amino acids (branched chain amino acid (BCAA): leucine, isoleucine, and valine) (28, 29). Therefore, we next assessed whether BCAAs are affected by YBX3 depletion in mouse skeletal muscle cells during differentiation. We found that YBX3 KD reduces the levels of BCAAs in proliferating and differentiating myoblasts (Fig. 6*A*). Taken together, these results suggest that YBX3 depletion specifically alters the intracellular levels of amino acids that are direct substrates of SLC1A5 (*i.e.*, glutamine) or indirectly affected by SLC1A5 (*i.e.*, BCAA) through coupled transport, which impacts nutrient availability for these cells (Fig. 6*C*).

### YBX3 depletion impacts skeletal muscle proliferation and differentiation

Amino acid availability is critical during differentiation in many cell types including embryonic stem cells (30), T-cells (31) myocytes (32), and YBX3 has been shown to affect cell proliferation and differentiation (10, 24). To begin to assess if YBX3 posttranscriptional regulation of AA transporters is critical for skeletal muscle differentiation, we investigated the proliferation rate of C2C12 during differentiation when YBX3 is depleted. To do this, we counted the number of live cells in YBX3 KD compared to control KD or nontransfected cells (C2C12). In YBX3 depleted cells, there was a significant reduction in cell number measured at all time points tested (Fig. 6*B*). The rate of cell proliferation was reduced in YBX3 depleted cells compared to the controls, specifically between one day and three days post initiation of differentiation (Fig. 6*B*). Importantly, the amount of dead or dying cells was consistent between the three conditions (data not shown). These results suggest that YBX3 depletion does not increase cell death, but rather slows cellular proliferation during differentiation.

Next, we assessed if YBX3 depletion impacts skeletal muscle differentiation. To do this, we examined the protein expression of early (*i.e.*, MYOD) and late (*i.e.*, MYOG and MYH1) differentiation markers in cells depleted of YBX3 across differentiation (Fig. 6*C*). In YBX3 depleted cells, the reduction of MYOD was delayed, while the induction of MYOG and MYH1 was markedly reduced compared to controls (Fig. 6*C*). Both YBX3 and SLC1A5 protein expression was reduced compared to control KD cells throughout differentiation (Fig. S6*B*). Of note, there is no change in the *MYOD*, *MYOG*, and *MYH1* mRNA levels observed in the RNAseq dataset (Fig. 1 and Table S1), which suggests that YBX3 depletion does not impact these mRNAs. Taken together, these data suggest that YBX3 expression is needed for both skeletal muscle proliferation and differentiation.

As YBX3 controls intracellular glutamine levels by posttranscriptional control of *SLC1A5* mRNA, we asked if cells starved of glutamine show similar effects on proliferation and differentiation as the YBX3 depleted cells. Not surprisingly, C2C12 cells starved of glutamine have decreased cell number and proliferation rate during differentiation (Fig. 6*B*), and neither late differentiation marker (MYOG or MYH1) was detected (Fig. 6*D*). The early differentiation marker, MYOD, had similar expression regardless of glutamine starvation, which could be due to MYOD protein expression before the onset of starvation (Fig. 6*C*). These data agree with previous findings showing glutamine is critical for skeletal muscle proliferation and differentiation (23, 33, 34, 35). The combined data suggest that YBX3 regulation of *SLC1A5* mRNA may be necessary to maintain the intracellular glutamine levels required for skeletal muscle cells to proliferate and differentiate (Fig. 6*E*).

## Discussion

Here, we report that the RNA-binding protein (RBP) YBX3 posttranscriptionally controls the mRNA of the amino acid transporter SLC1A5 (21), which in turn impacts intracellular amino acid levels during skeletal muscle differentiation. YBX3 depletion in C2C12 skeletal muscle cells significantly reduces the levels of *SLC1A5* mRNA levels during skeletal muscle differentiation (Figs. 1 and 2A) resulting in a decrease of SLC1A5 protein expression (Fig. 2*B*). Further, YBX3 directly binds the *SLC1A5* mRNA (Fig. 4), and the *SLC1A5* 3′ UTR is sufficient for YBX3 to regulate SLC1A5 expression in both proliferating and differentiating skeletal muscle cells (Fig. 5). YBX3 regulation of *SLC1A5* influences the intracellular levels of glutamine and BCAA during differentiation (Fig. 6*A*), and YBX3 depletion or glutamine starvation impairs skeletal muscle proliferation and differentiation (Fig. 6, *B*–*D*). In addition, we find that YBX3 and SLC1A5 protein expression strikingly increase during skeletal muscle differentiation, but there is no coinciding increase in mRNA expression for either transcript (Fig. 3). Taken together, these data suggest that YBX3 posttranscriptionally controls nutrient availability *via* direct control of the *SLC1A5* amino acid transporter mRNA, which may be needed to maintain intracellular glutamine levels necessary for proliferation and differentiation.

YBX proteins are known to broadly bind RNAs, which presents challenges to identify RNAs that are not only bound but regulated by YBX proteins (7, 36, 37). Our results reveal that YBX3 posttranscriptional control of amino acid transporter mRNAs occurs in both human and mouse cells; however, the SLC mRNAs regulated are distinct depending on the species and cell line (Fig. 2). Based on our previous eCLIP data in HeLa cells, YBX3 binds *SLC1A5* mRNA within the 3′ UTR (14); however, YBX3 does not regulate *SLC1A5* in HeLa or any of the other human cells tested here (Fig. 2). Interestingly, we find that *SLC7A5* and *SLC3A2* mRNAs are bound by YBX3 in mouse C2C12 cells (Fig. S4); however, these mRNAs are not significantly altered in YBX3 depleted C2C12 cells (Figs. 1 and 2A). RBPs, like YBX3, often form complexes with other proteins and regulatory RNAs on target mRNAs, collectively known as messenger ribonucleoprotein particles (mRNPs) (38, 39, 40). The make-up of these mRNPs is determined by a complex set of determinants including regulatory elements within the mRNA, modifications of either the mRNA or binding factors, and cellular context or expression (38, 39, 40). To assess potential differences in mRNA regulatory elements, we examined differences in the 3′ UTR sequence between species. Our analysis indicates that the 3′ UTR of *SLC1A5* is ∼52.5% and *SLC7A5* is ∼41.9% identical between mouse and human transcripts, and that the length and sequence of the 3′ UTRs are highly conserved across eukaryotes (41). Therefore, one intriguing possibility is that YBX3 binds these *SLC* mRNAs in all cellular contexts but the composition of the mRNP complex varies from humans to mice resulting in altered regulation; for example, a protein partner that regulates *SLC1A5* in mice is not expressed in humans and vice versa for *SLC7A5/SLC3A2* mRNAs in C2C12 cells. Further investigation into the mRNA regulatory elements and mRNP complexes formed on these mRNAs within different cellular contexts will help characterize how YBX3 binding in one context results in regulation while interaction in another context does not.

Our results highlight YBX3 as an RBP that directly binds and regulates *SLC1A5* during skeletal muscle cell differentiation. One observation for both YBX3 and SLC1A5 expression is the dramatic increase in protein expression without a concomitant increase in either transcript. (Fig. 3). In fact, our data show *SLC1A5* significantly decreases while its protein levels increase at later time points in differentiation (Fig. 3). This suggests there may be additional mechanisms to promote SLC1A5 protein expression, *e.g.*, translational control or protein stability. Additional experiments are needed to determine if these regulatory controls are YBX3-dependent or independent mechanisms. Lastly, it remains to be determined whether YBX3 regulation of intracellular amino acids is required for skeletal muscle proliferation and differentiation. The reduced rate of proliferation and impaired differentiation when YBX3 is depleted suggests that YBX3 is necessary for these processes. Glutamine is also critical for these processes, as cells starved of glutamine have drastic effects on proliferation and differentiation. As YBX3 regulates the mRNA of the glutamine transporter SLC1A5, this provides a potential link between YBX3 posttranscriptional control and these cellular processes. However, as YBX3 regulates other transcripts during differentiation, the effects on proliferation and differentiation could be due to reduced amino acid levels, regulation of other processes, or a combination of the varied processes YBX3 regulates.

We set-out to identify genes that are posttranscriptionally regulated by YBX3 in skeletal muscle. We focused on SLC1A5 due to its role in amino acid import, and our recent results linking YBX3 regulation to amino acid homeostasis in human cells (14, 21, 25, 28). However, these data reveal other potential YBX3 regulated genes in mouse skeletal muscle (Fig. 1 and Table S1). Gene ontology analysis did not reveal any obvious related biological processes or molecular functions between the genes with altered expression in YBX3 depleted cells. Genes with decreased expression upon YBX3 depletion included three dehydrogenases (Nad(P)H dehydrogenase, quinone 1 (NQO1) (42, 43), glucose-6-phosphate dehydrogenase X-linked (G6PDX) (44), and phosphogluconate dehydrogenase (PGD) (45)), which further links YBX3 regulation to cellular energy. Multiple collagen type (COL10A1, COL6A4, COL5A1, and COL5A2) mRNAs were increased in expression in YBX3 depleted cells. Collagen is the most abundant component of skeletal muscle extracellular matrix providing critical functions in tissue elasticity, contractile force, differentiation, and growth. Interestingly, KD of COL5A1 in mouse muscle stem cells resulted in an upregulation of differentiation markers (MYOD and MYOG) and for the cells to differentiate from a quiescent state (46). Consistent with these results, YBX3 depletion impairs differentiation and results in an increase of *COL5A1* mRNA. Future studies could examine if YBX3 regulation of collagen expression is critical for differentiation. Overall, a better understanding of the unknown relationship between YBX3 and collagen offers another potential line of investigation regarding YBX3 regulation (47). To fully characterize YBX3 posttranscriptional regulation in skeletal muscle cells, further research needs to be done to determine if YBX3 directly binds to regulate these mRNAs, or if the observed changes are due to indirect effects. Regardless, as SLC amino acid transporters are dynamically expressed and critical for skeletal muscle differentiation (19, 20, 21, 22, 23), understanding YBX3 posttranscriptional regulation of SLC mRNAs will provide insight into the regulatory networks governing this process.

## Experimental procedures

### Experimental model and cell culture

All mammalian cells (mouse and human origin) were grown at 37 °C and 5% CO2 in Dulbecco’s modified Eagle media (DMEM) (GIBCO, #R11965-092) with 10% fetal bovine serum (GIBCO, #R16000-044), 1% penicillin/streptomycin (GIBCO, #R15140-122, and 1% L-glutamine (GIBCO, #R25030-081) except HCT116 cells. HCT116 cells (human origin) were grown in McCoy’s 5A Medium (Sigma-Aldrich, M8403), with all other growth conditions consistent with the conditions used for the other cell lines. To induce differentiation, differentiation media (DMEM with 10% Horse Serum (GIBCO, #26050088), 1% penicillin/streptomycin and 1% L-glutamine) was added to the cells 1 day after siRNA transfection. Cells were harvested 24, 72, and 120 h after inducing differentiation.

### SiRNA reverse transfection

Cells were grown until approximately 80% confluent, trypsinized (0.05%, #R25300-054, GIBCO) and counted (Bio-Rad TC20). Approximately 200,000 cells were seeded into each well in a 6-well plate for reverse transfections following the manufacturer’s recommendations (Lipofectamine RNAiMax, Invitrogen, #137780755). Control and YBX3 siRNAs were used from Dharmacon (all siRNA sequences are located in Table S2). Cells were harvested 48, 96, and 144 h after transfection depending on the experiment. Control conditions included cells transfected with nontargeting siRNAs (control KD; C2C12, HCT116, HepG2, and NIH3T3), and nontransfected cells (C2C12).

### Sample preparation for RNaseq

High-throughput library preparation using Illumina stranded mRNA kit (#20040534). RNA integrity was assessed with Agilent bioAnalyzer. Barcoded and stranded RNaseq libraries were prepared from high-quality total RNA samples (500 ng/sample) using the Illumina TruSeq RNA Sample Preparation v2 Kit. Obtained libraries that passed the quality control step were pooled in roughly equimolar amounts then sequenced on a NovaSeq 6000 SP flowcell to 100 bp using 10 × 10 bp indexing reads to read the barcodes generating 51 million reads (MR) ± 12 MR per sample, with a minimum of 37.44 MR of approximately 85 nucleotides each.

### RNaseq data analysis

The reads were aligned with SALMON for mapping directly to the genome to the GRCM39 genome using Gencode M30 (canonical list) with a mapping efficiency of 80 to 90%. Significantly expressed genes were determined with DESeq2 (48) comparing YBX3 KD samples against control KD (absolute fold change >1.3, p-adj <0.001) with independent hypothesis weighting for false discovery rate control under multiple hypothesis testing (49).

### RNA harvesting and purification

One well of each condition was maintained according to cell culture protocol in a 6-well plate. Cells were harvested at the indicated time points: 0D (48 h after siRNA transfection), 1D postdifferentiation 48 h after siRNA transfection, but differentiated), and 3D postdifferentiation (4 days after siRNA transfection). The adherent cells were washed with PBS, and then scraped in 500 μl of PBS twice. They were then collected in a microfuge tube and spun at 1000 relative centrifugal force for 5 min. The supernatant was removed and the cell pellets were stored at −80 °C. RNA was purified using the GeneJET RNA purification kit (cat#K0731, Thermo Fisher Scientific), and the RNA quality was assessed through gel electrophoresis.

### Reverse transcription quantitative PCR

Five hundred nanograms of total RNA harvested was used to synthesize complementary DNA (cDNA) using Superscript III reverse transcriptase from the First Strand cDNA Synthesis kit (cat#18080051, Thermo Fisher Scientific). Quantitative polymerase chain reaction (RT-qPCR) was performed using Sybr-Green qPCR Master Mix (cat# 4309155, Applied Biosystems) using primers listed in Table S2 or in previously reported research (14). All experiments were normalized to the housekeeping genes *UBN1* for mouse cells and *GUSB* for human cells.

### Branched chain amino acid, L-Alanine and L-glutamine assays

C2C12 cells were grown in accordance to the conditions outlined above. Once confluent, cells were seeded into 6-well plates and transfected. Twenty-four hours following reverse transfections, differentiation was induced in designated wells. After the differentiation period, cells were washed with 1x PBS, trypsinized, resuspended in DMEM (cat# R11965-092, GIBCO), and counted (Bio-Rad TC20). Cells were then spun down, supernatant was removed, and pellets were resuspended in 1x PBS. Amino acid concentrations were determined using the Bioassay Systems EnzyChrom Glutamine Assay Kit (EGLN-100, BioAssay Systems), Abcam BCAA Kit (cat# ab83374, ABCAM) and the Abcam L-Alanine Assay Kit (cat# ab83394, ABCAM) according to the manufacturer’s recommendations using the Tecan F200 Colorimetric Microplate reader. Absorbance was measured at 570 nm (glutamine and L-alanine) and 450 nm (BCAA) in a Corning Costar 96-Well, Cell Culture-Treated, Flat-Bottom Microplate.

### Data analysis

Microsoft Excel was used to organize the data output for all experiments. The control and experiment C_T_ values were compiled and compared using the ΔΔCt method. Threshold cycle (C_T_) values (measurements of the number of replications to reach certain values) were used to assess the copies of target mRNA transcripts of YBX3. Measured mRNA levels were compared relative to the value of the nontargeting (control KD) samples of the corresponding day of differentiation. For the BCAA and L-alanine measurements, cell counts were used to standardize amino acid measurements. Prism was used to create plots, error bars, and statistical analysis. All RT-qPCR data and amino acid quantifications are displayed as single points, mean ± SD. A Student’s *t* test was used to determine significance in values for control *versus* YBX3 KD siRNA samples comparing the ΔCt values from different biological replicates for each condition.

### Coimmunoprecipitation assay

C2C12 cells were grown until 70 to 80% confluent, after which differentiation was induced using 10% horse serum in complete media. Twenty-four hours later, the differentiated cells were collected by scraping in PBS and centrifuged to collect a cell pellet. About 20 million cells were collected for each IP sample. Cells were lysed using 50 mM Tris–HCl (pH 7.4), 150 mM NaCl, 1% NP-40, 5 mM EDTA (pH 8.0), 0.1% SDS, 1× Protease Inhibitor cOmplete Mini EDTA-free (Sigma-Aldrich, cat# 11836170001), and rRNAsin (Promega, cat# 21586406), and then centrifuged at 4 °C for 10 min at 10,000*g*. IP was performed using Dynabeads Protein A (Novex, cat# 10001D) with 3 μg of the following antibodies: ZONAB (YBX3) (Bethyl, cat# A303-070A) and Rabbit IgG (Bethyl, cat# P120-201). The supernatant was saved for analysis. The beads were washed with 50 mM Tris–HCl (pH 7.4), 500 mM NaCl, 1% NP-40, 5 mM EDTA (pH 8.0), 0.1% SDS, 0.5% Triton-X 100, 1X Protease Inhibitor. The beads were then eluted in 1X PBS, 0.02% Tween-20 (pH 7.4), 1X Protease Inhibitor. Twenty percent of the volume was saved for immunoblotting protein analysis, while the remaining 80% was saved for RNA precipitation.

### End-point PCR

RNA was extracted and purified from the input and supernatant samples using the GeneJET RNA Purification Kit (Thermo Fisher Scientific, cat# K0731). RNA was extracted from IP samples through phenol-chloroform extraction. The first extraction was performed using 25:24:1 Phenol:Chloroform:Isoamyl (Thermo Fisher Scientific, cat# am9730), and the second extraction was performed using ≥99.8% chloroform (J. T. Baker, cat# UN1888). Reverse transcription was performed using the SuperScript III kit (Invitrogen, cat# 18080044) to create cDNA. PCR was performed on this cDNA using Q5 Hotstart High-Fidelity polymerase (NEB, cat# m0949S) and primers specific to *SLC7A5*, *SLC3A2*, and *SLC1A3*, as well as the housekeeping gene *UBN1*. For *SLC7A5* and *SLC3A2*, the PCR of the input and supernatant samples was performed with 26 cycles. For all other samples, including the IP and No RT samples of *SLC7A5* and *SLC3A2* and all samples for *SLC1A3* and *UBN1*, 32 cycles were used. PCR amplification was visualized through agarose gel electrophoresis.

### Western blotting

Protein concentration of cell lysates was determined using a Bradford assay (Bio-Rad Protein Assay Dye, Bio-Rad, cat# 5000006) with absorbance at 595 nm measured using a Pharmacia LKB Ultrospec III UV/Vis spectrophotometer. Twenty-five micrograms of protein extracts from cell lysates were run on a 4 to 15% Tris-Glycine eXtended (TGX) gel (Mini-PROTEAN TGX gel, Bio-Rad, cat# 456083). The gel was transferred to a 0.2 μm nitrocellulose membrane (*Trans*-Blot Turbo Transfer Pack, Bio-Rad, cat# 1704158) using a Bio-Rad *Trans*-Blot Turbo Transfer System. The primary antibodies used in immunoblotting were anti-ZONAB (Bethyl, Cat# A303-070A, RRID:AB_10893576), 1:2000; anti-ACTIN clone C4 (Millipore, Cat# MAB1501, RRID:AB_2223041), 1:5000; anti-ASCT2 V501 (Cell Signaling, Cat# 5345, RRID:AB_10621427); 1:2000 to 1:10,000 depending on cell line, anti-LAT1 (Cell Signaling, cat# 5347, RRID:AB_10695104), 1:2000; and anti-CD98 (H-300) (Santa Cruz Biotechnology, cat# sc-9160, RRID:AB_638288), 1:10,000. Secondary antibodies used were TrueBlot Anti-Rabbit IgG horseradish peroxidase (HRP) (Rockland, cat# 18-8816-31), 1:10,000; Goat Anti-Rabbit IgG H&L HRP (Abcam, cat# ab205718), 1:10,000 and Goat Anti-Mouse IgG H&L HRP (Abcam, cat# ab205719), 1:10,000. ImageJ (https://imagej.nih.gov/ij/) was used to quantify the relative intensity of protein to the levels of the ACTB loading control on the Western blots in Figure 2, Figure 3*A*, and 6, *C* and *D* following the web-based tutorial: http://lukemiller.org/index.php/2010/11/analyzing-gels-and-western-blots-with-image-j/.

### Molecular cloning of NanoLuc reporter RNAs

pCDNA5-FRT-TO-NanoLucPEST MS2x6 (gift from Dr Michael D. Sheets, University of Wisconsin-Madison) was digested with restriction enzymes, NotI-HF (NEB cat# R3189S) and SwaI (NEB cat# R0604S), following protocols as described by NEBCloner. All inserts were amplified from C2C12 cDNA. Primers (Table S2) were designed against the following RefSeq: SLC1A5 NM_009201.2, SLC7A5 NM_011404.3, SLC3A2 NM_001161413.1 and SLC1A3 NM_148938.3. All PCR-amplified inserts were each cloned into the NotI and SwaI sites of pcDNA5-FRT-TO-NanoLucPEST MS2x6 vector *via* Gibson Assembly. All assembled plasmids were confirmed *via* Sanger sequencing (GENEWIZ from Azenta Life Sciences). Of note, the SLC7A5 3′ UTR contained only the second half of the SLC7A5 3′ UTR, sequence coordinates: nucleotide 947 to nucleotide 1894.

## Data availability

All data that supports the findings of this study can be found within the manuscript and Supporting information except for the raw RNAseq data. The raw RNAseq data can be found at GEO: GSE240445.

## Supporting information

This article contains supporting information.

## Conflict of interest

The authors declare that they have no conflicts of interest with the contents of this article.
