# Supplementary material for: The YBX3 RNA-binding protein posttranscriptionally controls SLC1A5 mRNA in proliferating and differentiating skeletal muscle cells
Source: J Biol Chem. 2023 Dec 29;300(2):105602. doi: 10.1016/j.jbc.2023.105602 (PMC10837625; doi:10.1016/j.jbc.2023.105602)
Supplement: Supporting Figures S1–S6 [file mmc1.pdf]

Supplemental Information for:

The YBX3 RNA-binding protein posttranscriptionally controls *SLC1A5* mRNA in proliferating and differentiating skeletal muscle cells

Silina Awad^, William Skipper^, William Vostrejs^,[2], Kendall Ozorowski[3], Kristen Min[4], Liva Pfuhler[5], Darshan Mehta and Amy Cooke[1,6*]

[1]Haverford College, 370 Lancaster Ave, Haverford PA 19041, USA

[2]New Address: University of Pennsylvania, 421 Curie Blvd, Philadelphia PA 19104, USA

[4]New Address: Sidney Kimmel Medical College, Thomas Jefferson University, 1025 Walnut St, Philadelphia PA 19107, USA

[4]New Address: Children's Hospital of Philadelphia, 3401 Civic Center Blvd, Philadelphia PA 19104, USA

[5]New Address: Division of Gastroenterology, Hepatology and Nutrition, Cincinnati Children's Hospital Medical Center, Cincinnati OH 45229, USA

[6]Lead Contact

^ These authors contributed equally to this work.

*Correspondence: acooke1@haverford.edu

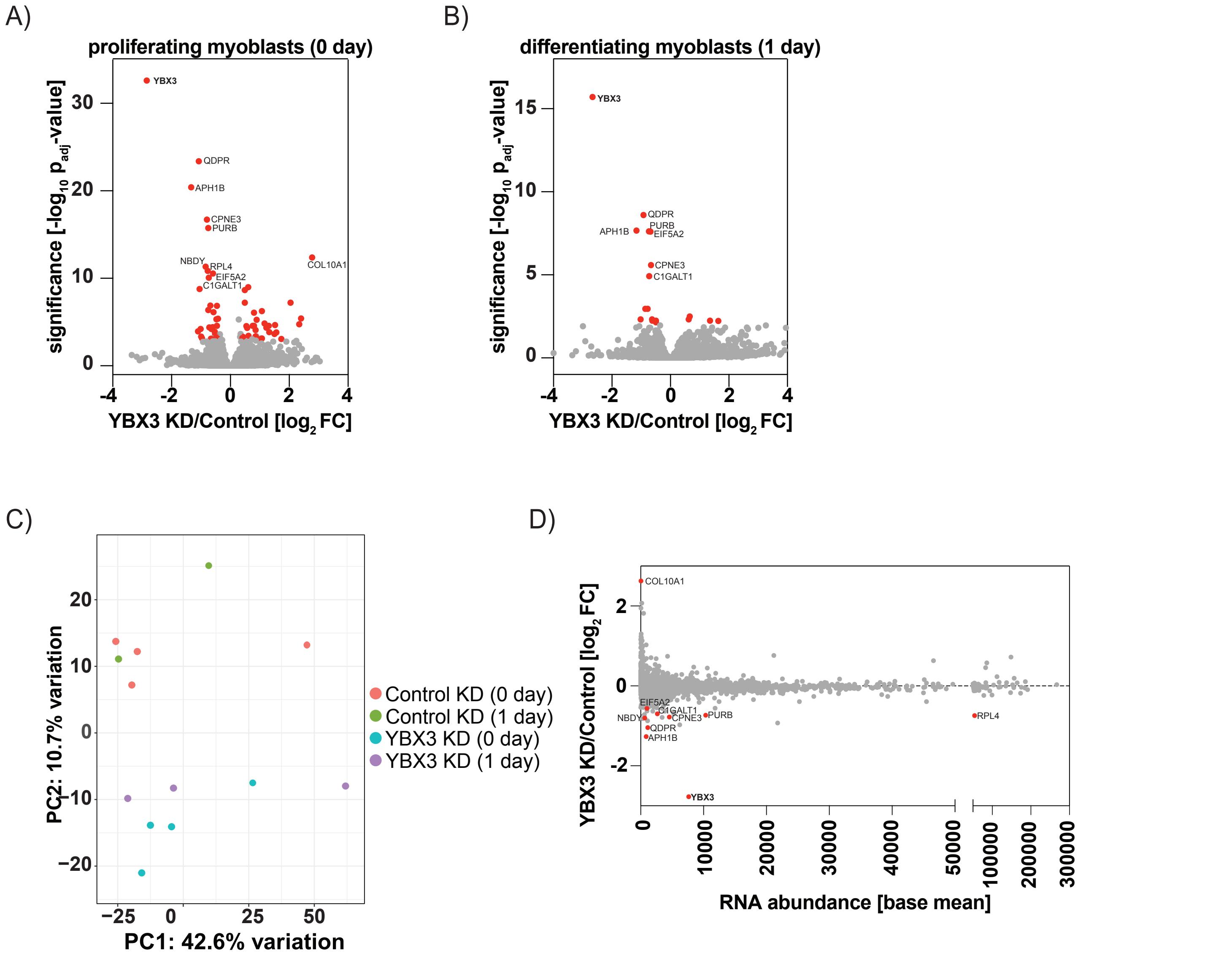

Figure S1, related to Figure 1: Transcriptomic Changes Elicited by YBX3 Knock Down in Skeletal Muscle Cells. A & B) Volcano plot of altered transcripts from proliferating (A) or differentiating (B) myoblasts. RNAs were classified as significant (FC > +/-1.3 and p-adjusted 0.001; red dots) or non-significant (gray dots). Labels of significantly altered transcripts with a p-adj of 0.0001 indicated. C) Principle component analysis (PCA) for RNAseq libraries generated from cells treated with YBX3 (YBX3 knock-down (KD)) or control (Conrol KD) siRNAs. D) RNA abundance versus log2 FC for changes in RNA levels of cells transfected with siRNAs to YBX3 versus control. RNAs were color coded as in (A) & (B).

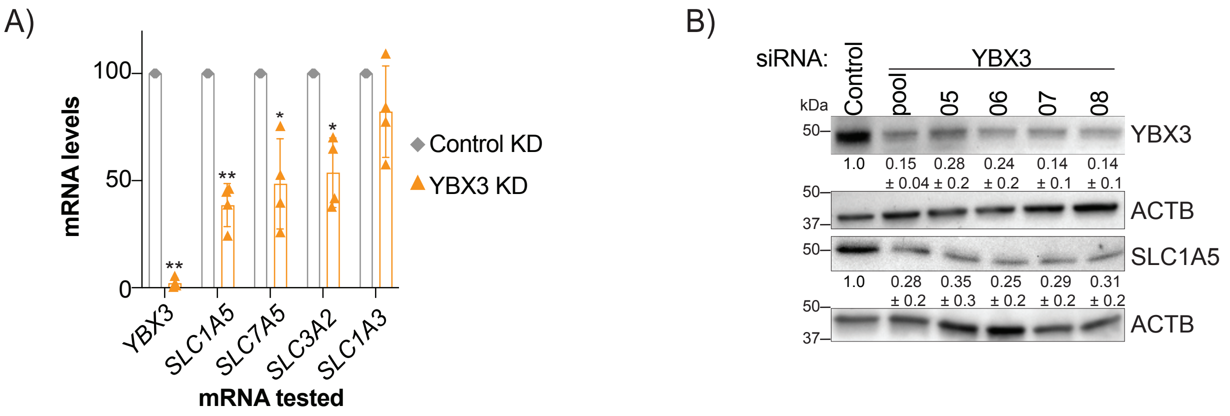

Figure S2, related to Figure 2: YBX3 depletion reduces SLC1A5 expression in C2C12 and NIH3T3 mouse cells but not in HeLa, HepG2 or HCT116 human cell lines. A) Reverse transcription quantitative polymerase chain reaction (RT-qPCR) of relative mRNA levels after YBX3 KD versus control KD in C2C12 cells before differentiation initiation (i.e., proliferating myoblasts). **p < 0.01 and *p < 0.05 with paired Student's t-test, n=>3. All RT-qPCR values normalized to *UBN1* mRNA levels. B) Immunoblot analysis of protein levels for YBX3 and SLC1A5 along with actin loading control (ACTB) in cells transfected with single YBX3 siRNAs (05, 06, 07, & 08) versus the YBX3 pool and control siRNAs.

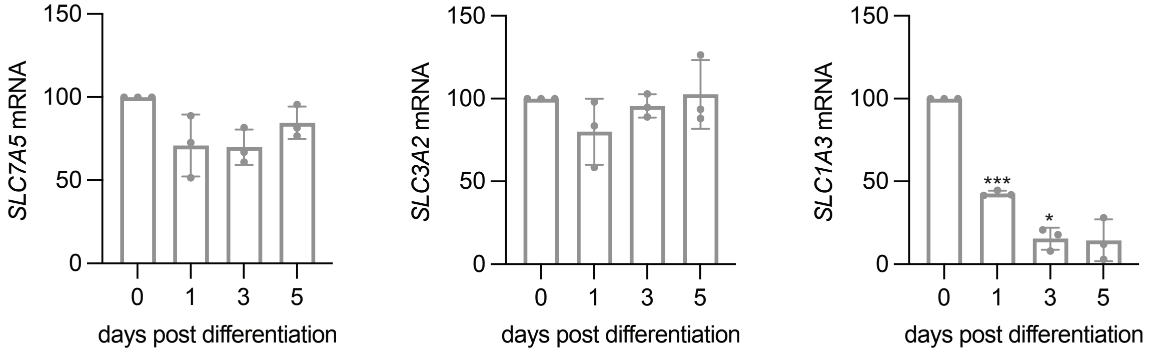

Figure S3, related to Figure 3: YBX3 and SLC1A5 protein expression increases throughout differentiation independent of changes in mRNA. RT-qPCR of *SLC7A5*, *SLC3A2*, and *SLC1A3* relative mRNA levels across 5 days of differentiation. ***p < 0.001 and *p < 0.05 with paired Student's t-test, n=> 3. RT-qPCR values normalized to *UBN1* mRNA levels.

A)

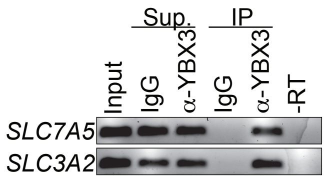

B)

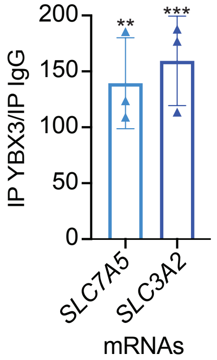

Figure S4, related to Figure 4: YBX3 interacts with *SLC1A5* mRNA in skeletal muscle cells
A) Endpoint PCR of indicated mRNAs from each sample in (Figure 2A), n=>3. B) RT-qPCR of indicated mRNA from IP samples plotted as a ratio of YBX3 IP/Control IP. RT-qPCR values normalized to input mRNA levels. ***p < 0.001 and **p < 0.01 with paired Student’s t-test, n=3.

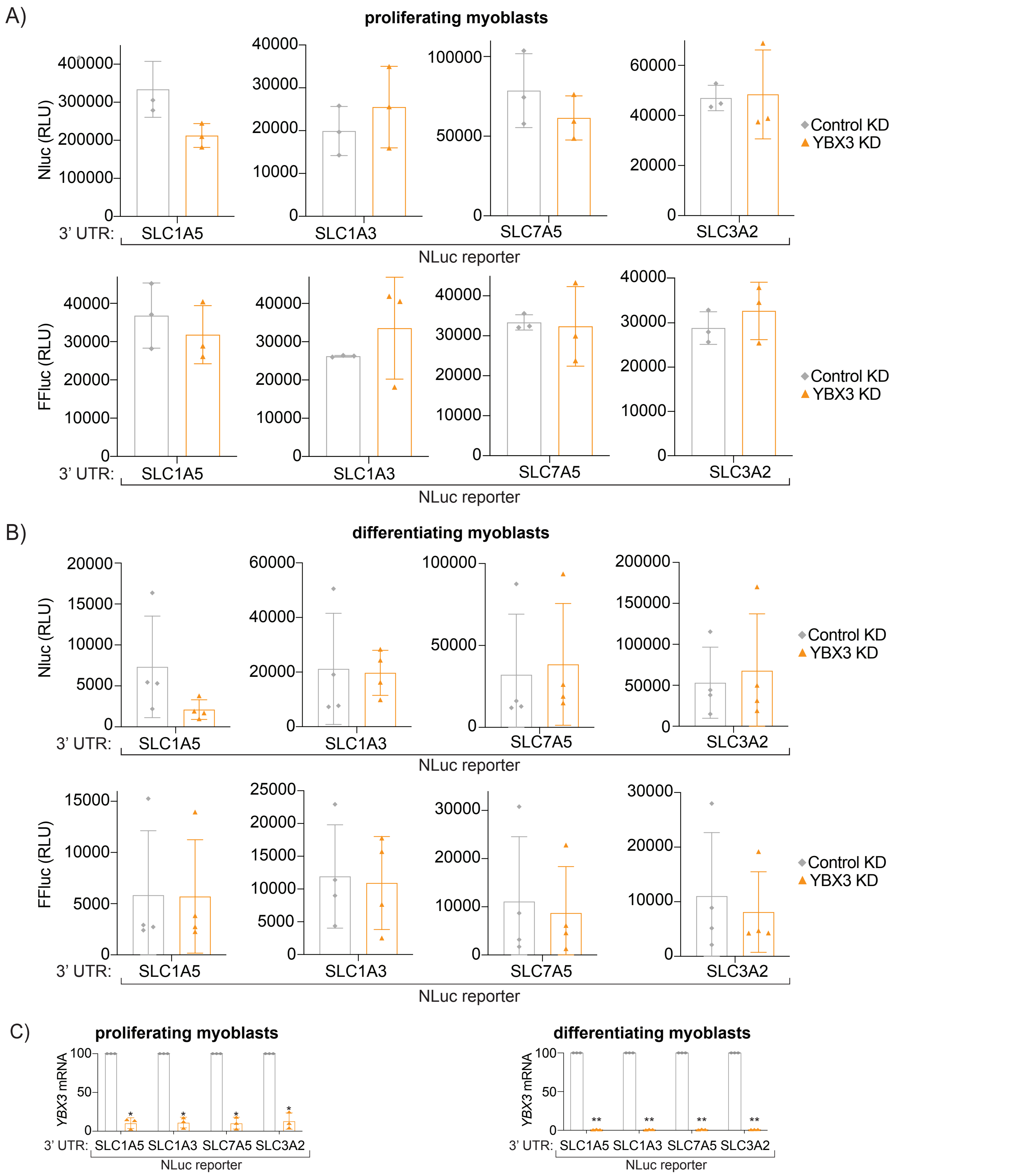

Figure S5, related to Figure 5: *SLC1A5* 3' UTR is sufficient for YBX3 regulation A & B) luciferase assay of NanoLuc (NLuc; top) or firefly (FFLuc; bottom) reporter expression with the indicated SLC 3' UTRs (SLC1A5, SLC1A3, SLC7A5, and SLC3A2) in proliferating (A) or differentiating (B) cells. All luciferase assay data are displayed as single points, mean ± SD. n>3. C) RT-qPCR of YBX3 mRNA levels after YBX3 KD versus control KD in proliferating (left) or differentiating (right) transfected luciferase samples. **$p < 0.01$ and *$p < 0.05$ with paired Student's t-test, n>3.

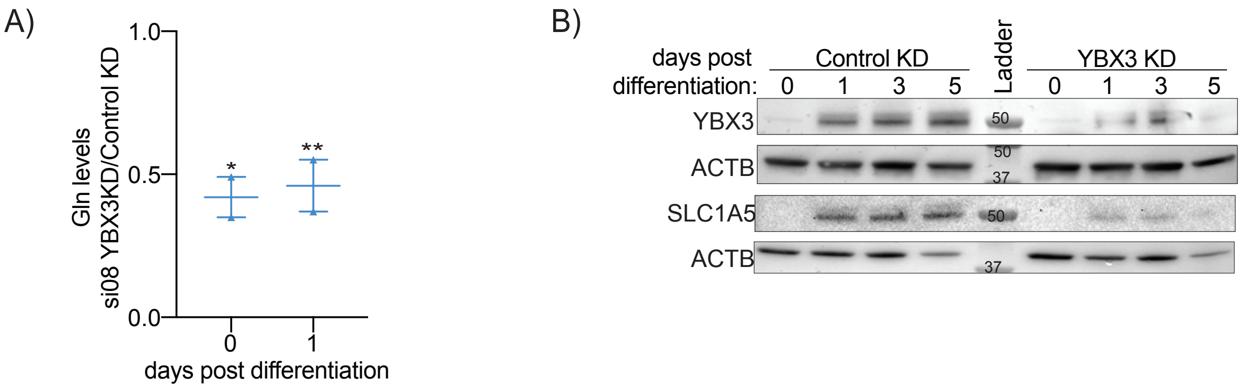

Figure S6, related to Figure 6: YBX3 depletion specifically reduces intracellular glutamine and inhibits cell proliferation and differentiation. A) Relative concentration of glutamine (Gln) in C2C12 cells transfected with the single si08 YBX3 (YBX3 KD) or non-targeting siRNA (control KD) before (0 day) or 1-day post-differentiation (1-day). Box plot displays standard deviation and the line marks the median value for the replicates. Each point indicates a biological replicate, n = 2 and $^{**}p < 0.01$ and $^{*}p < 0.05$ with paired Student's t-test. B) Immunoblot analysis of protein levels for YBX3 and SLC1A5 expression along with actin loading control (ACTB) in cells transfected with non-targeting (control KD) or YBX3 targeting (YBX3 KD) siRNAs at 0,1,3 and 5-days post differentiation.
