# Supplementary material for: The YBX3 RNA-binding protein posttranscriptionally controls SLC1A5 mRNA in proliferating and differentiating skeletal muscle cells
Source: J Biol Chem. 2023 Dec 29;300(2):105602. doi: 10.1016/j.jbc.2023.105602 (PMC10837625; doi:10.1016/j.jbc.2023.105602)
Supplement: Supporting Tables S1 and S2 legend [file mmc4.pdf]

Supplemental Table 1: RNAseq analysis with Deseq2. Mapped reads from YBX3 KD were compared to mapped reads from Control KD samples to determine significantly expressed genes. *Tab 1*: Description. *Tab 2*: YBX3 KD vs Control KD tab: YBX3 KD vs Control KD for all genes identified between proliferating myoblasts (0 day) and differentiating myoblasts (1 day). *Tab 3*: 0 day YBX3 KD vs Control KD tab: YBX3 KD vs Control KD for all genes identified between proliferating myoblasts (0 day). *Tab 4*: 1 day YBX3 KD vs Control KD tab: YBX3 KD vs Control KD for all genes identified between differentiating myoblasts (1 day). *Tab 5*: 0 day vs 1 day Control KD: 0 day vs 1 day for all genes identified between the two timepoints in Control KD. *Tab 6*: Decreased padj .001 log2FC<-0.4 tab: significantly altered genes with reduced expression in YBX3 KD vs Control KD. *Tab 7*: Increased padj .001 log2FC<0.4 tab: significantly altered genes with increased expression in YBX3 KD vs Control KD.

Supplemental Table 1: Primers and siRNAs used in study. *Tab 1*: qPCR & endpoint PCR primers: List of primer sequences for qPCR and endpoint PCR analysis. *Tab 2*: Cloning primers gene sequences: List of primer sequences for cloning the 3' UTRs of indicated genes. *Tab 3:* siRNA sequences: List of siRNAs used in the study including non-targeting controls and human and muse YBX3.
